# The Murakami Cohort Study of vitamin D for the prevention of musculoskeletal and other age-related diseases: a study protocol

**DOI:** 10.1186/s12199-018-0715-2

**Published:** 2018-06-26

**Authors:** Kazutoshi Nakamura, Ribeka Takachi, Kaori Kitamura, Toshiko Saito, Ryosaku Kobayashi, Rieko Oshiki, Yumi Watanabe, Keiko Kabasawa, Akemi Takahashi, Shoichiro Tsugane, Masayuki Iki, Ayako Sasaki, Osamu Yamazaki

**Affiliations:** 1 Division of Preventive Medicine, Niigata University Graduate School of Medical and Dental Sciences, 1-757 Asahimachi-dori, Chuo-ku, Niigata, 951-8510 Japan; 2 Department of Food Science and Nutrition, Nara Women’s University Graduate School of Humanities and Sciences, Kitauoyahigashimachi, Nara, 630-8506 Japan; 3 Department of Health and Nutrition, Niigata University of Health and Welfare, 1398 Shimami-cho, Kita-ku, Niigata, 951-3198 Japan; 4 Department of Physical Therapy, Niigata University of Health and Welfare, 1398 Shimami-cho, Kita-ku, Niigata, 951-3198 Japan; 5 Department of Rehabilitation, Niigata University of Rehabilitation, 2-16 Kaminoyama, Murakami, Niigata, 958-0053 Japan; 6 Department of Health Promotion Medicine, Niigata University Graduate School of Medical and Dental Sciences, 1-757 Asahimachi-dori, Chuo-ku, Niigata, 951-8510 Japan; 7 Center for Public Health Sciences, National Cancer Center, 5-1-1 Tsukiji, Chuo-ku, Tokyo, 104-0045 Japan; 8 Department of Public Health, Kindai University Faculty of Medicine, 377-2 Oonohigashi, Osaka-Sayama, Osaka, 589-8511 Japan; 9 Murakami Public Health Center, 10-15 Sakanamachi, Murakami, Niigata, 958-0864 Japan; 10 Niigata Prefectural Office, 4-1 Shinkocho, Chuo-ku, Niigata, 950-0965 Japan

**Keywords:** Cohort studies, Dementia, Musculoskeletal diseases, Osteoporotic fractures, Vitamin D

## Abstract

**Background:**

Age-related musculoskeletal diseases are becoming increasingly burdensome in terms of both individual quality of life and medical cost. We intended to establish a large population-based cohort study to determine environmental, lifestyle, and genetic risk factors of musculoskeletal and other age-related diseases, and to clarify the association between vitamin D status and such diseases.

**Methods:**

We targeted 34,802 residents aged 40–74 years living in areas of northern Niigata Prefecture, including Sekikawa Village, Awashimaura Village, and Murakami City (Murakami region). The baseline questionnaire survey, conducted between 2011 and 2013, queried respondents on their lifestyle and environmental factors (predictors), and self-reported outcomes. Plasma 25-hydroxyvitamin D (25[OH]D) concentration, an indicator of vitamin D status, was determined with the Liaison® 25OH Vitamin D Total Assay. The primary outcome of this study was osteoporotic fracture; other outcomes included age-related diseases including knee osteoarthritis, perception of chronic pain, dementia, and long-term care insurance use. Mean ages of men and women were 59.2 (SD = 9.3, *N* = 6907) and 59.0 (SD = 9.3, *N* = 7457) years, respectively. From the blood samples provided by 3710 men and 4787 women, mean 25(OH)D concentrations were 56.5 (SD = 18.4) nmol/L (22.6 ng/mL) and 45.4 (SD = 16.5) nmol/L (18.2 ng/mL), respectively.

**Discussion:**

Follow-up surveys are planned every 5 years for 15 years, and incident cases of our targeted diseases will be followed at hospitals and clinics in and nearby the cohort area. We anticipate that we will be able to clarify the association between vitamin D status and multiple disease outcomes in a Japanese population.

## Background

The average life expectancy in Japan is one of the highest in the world. This is attributed in part to successful prevention and treatment of fatal diseases, such as cancer, cardiovascular disease, and cerebrovascular disease. Consequently, ageing is accelerating. In 2016, 27.3% of the population comprised elderly persons over 65 years, and that in 2055 is projected to be as high as 38.0% [1]. In such an ageing society, common age-related diseases, such as musculoskeletal diseases, become highly burdensome in terms of both individual quality of life and medical cost. For example, osteoporotic fracture and knee osteoarthritis impair physical function, leading to decreased levels of activities of daily living (ADL), and eventually to higher numbers of elderly individuals with physical disabilities requiring care. The National Livelihood Survey of the Ministry of Health, Labour and Welfare in Japan [1] reported that fractures and knee osteoarthritis account for 10.2 and 10.9%, respectively, of all cases of disability requiring care, and the sum of these (21.1%) is comparable to the percentage of individuals with cerebrovascular diseases (21.5%) and dementia (15.3%). Furthermore, musculoskeletal disorders are a main cause of chronic pain, which is also becoming a major public health concern [2].

The economic burden on medical insurance companies from musculoskeletal and other age-related diseases causing disability has been enormous. For example, the annual cost of treatment and after-care for hip and vertebral fractures in Japan has been estimated as 750 and 150 billion yen (nearly equivalent to 7.5 and 1.5 billion dollars), respectively [3]. Rapid increases in the economic burden on long-term care insurances are also problematic in Japan. The cost of the long-term care insurance in 2000 was 3.6 trillion yen, but had over doubled this amount, to 9.8 trillion yen in 2015 [1]. Accordingly, prevention of age-related musculoskeletal diseases is a top priority at present and in the near future.

With regard to age-related diseases, vitamin D has received increasingly more attention, as its insufficiency is considered a potential risk factor for age-related bone diseases [4] and other age-related chronic diseases, such as some cancers, vascular diseases, diabetes, and dementia [5, 6].Vitamin D status is also of great interest because vitamin D insufficiency is widespread worldwide [7]. Although a number of cohort studies on vitamin D status and chronic diseases have been conducted in European and North American countries, large cohort studies that target East Asian populations are lacking [8].

Over the last several years, we have conducted medium-scale epidemiologic studies on vitamin D status, osteoporosis, and osteoporotic fractures in Japanese populations and reported that higher vitamin D concentrations are associated with higher bone mass and a lower incidence of osteoporotic fractures [8, 9]. Japanese people are an interesting population in terms of vitamin D nutrition because they frequently eat fish, a major source of vitamin D, and consume much more fish than populations in Europe and North America [10]. This implies that some lifestyles, including dietary habits, could impact vitamin D status and bone health. Comprehensive approaches, including genetic information, should be used to determine risk factors for osteoporotic fractures.

Against this backdrop, the present study aimed to establish a large population-based cohort study to determine (1) environmental, lifestyle, and genetic risk factors of osteoporotic fractures, and their interactions; (2) factors related to other age-related diseases, including knee osteoarthritis, chronic pain, dementia, disability, and some cancers; and (3) the impact of vitamin D status on the incidence of these diseases or disorders.

## Methods/design

### Participants

**Fig. 1:**
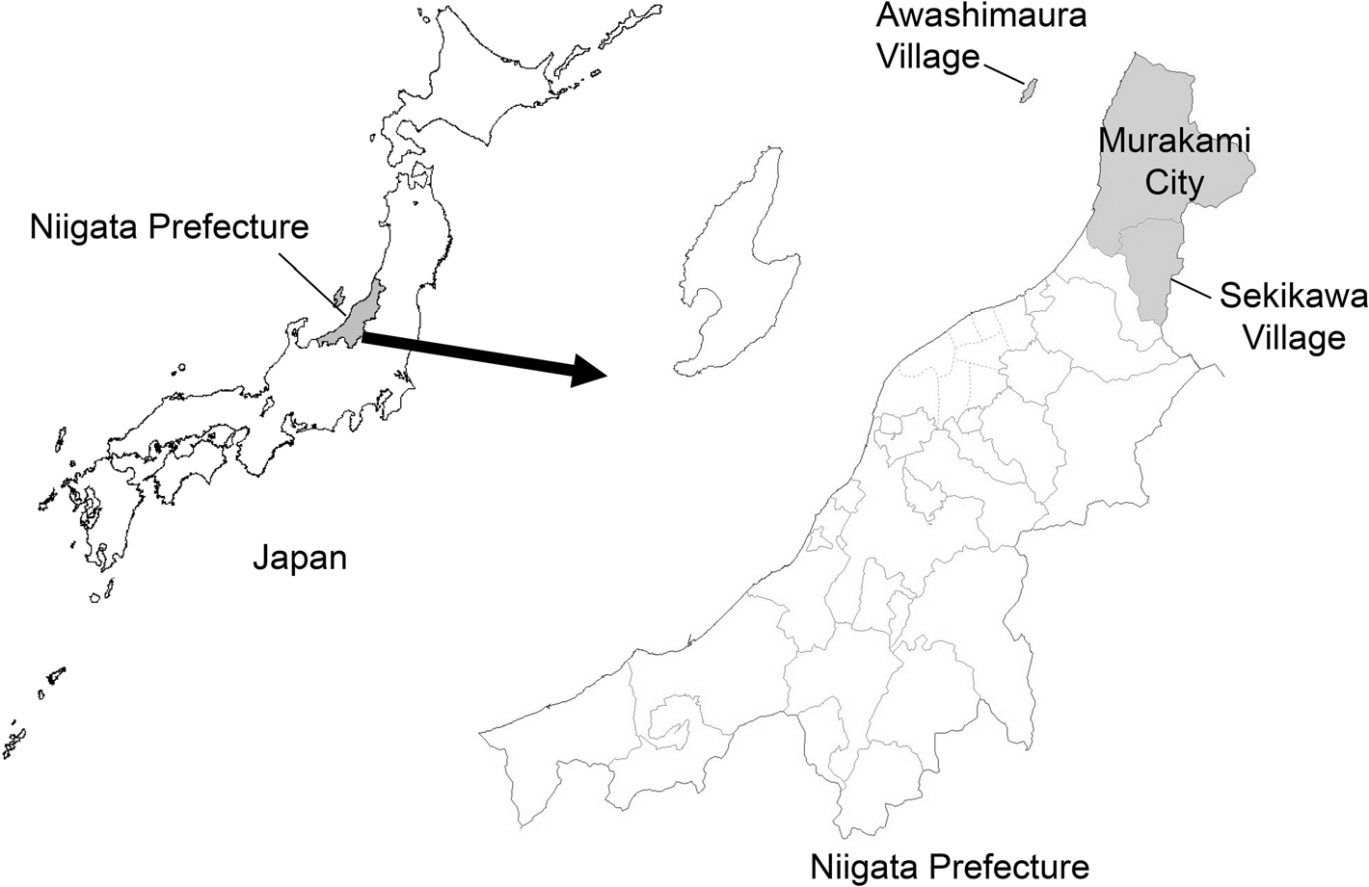
Location of Murakami City, Sekikawa Village, and Awashimaura Village in Niigata Prefecture, Japan

The Murakami Cohort Study is a population-based study of age-related musculoskeletal diseases that targeted individuals aged between 40 and 74 years living in areas of northern Niigata Prefecture under the jurisdiction of the Murakami Public Health Centre (Murakami region). These areas include Sekikawa Village (3065 residents on April 1, 2011), Awashimaura Village (178 residents on January 1, 2011), and Murakami City (31,559 residents on January 1, 2012) (Fig. 1). All 34,802 residents in the Murakami region aged between 40 and 74 years were invited to participate in the study. Of these, 14,364 (41.3%) participated in the cohort study, and 8497 of the 14,364 participants provided blood samples. Informed consent was obtained from all participants. The protocol of this study was approved by the Ethics Committee of Niigata University School of Medicine (No. 1324 for study design and 452 and 481 for genetic analysis).

### Baseline study

In the baseline survey, a self-administered questionnaire was distributed through a community-based communication network to Sekikawa residents and Awashimaura residents in early 2011 and to Murakami City residents in late 2011 and early 2012. Questionnaires were completed by those who agreed to participate in the study and collected through the network. A supplemental mail survey was used for communities where such a network is not available. At the time of the baseline survey, participants were asked to provide blood samples for future biochemical and genetic analyses. Blood sampling was performed only among those who provided consent between 2011 and early 2013 at one of the following settings: annual health check examinations provided by the local government, workplace health check examinations, local hospitals, clinics, and health care facilities of internal medicine, and an additional setting provided by our team. Blood samples were collected directly by our team, except at local hospitals, clinics, and health care facilities, where quality control of samples was ensured by respective clinical laboratories and samples were collected through BML, Inc. (Tokyo, Japan). Additional participants were recruited from annual health check examinations and workplace health check examinations between 2012 and 2013. In a subsample, clinical examination data of participants were collected from annual health check examinations provided by the local government or workplace health check examinations.

### Follow-up plans

Follow-up questionnaire surveys are planned every 5 years for 15 years, with the aim to reevaluate exposures, such as lifestyles, and obtain self-reported outcomes, such as chronic pain.

Most outcome measures, e.g., incident cases of target diseases, are thoroughly be followed every year for 20 years at relevant hospitals and clinics in the Murakami region and secondary or tertiary medical centres around the Murakami region (Niigata Prefectural Shibata Hospital and Tsuruoka Municipal Shonai Hospital). Incident cases of osteoporotic fractures and osteoarthritis are followed at hospitals and clinics involved in orthopaedics, including Murakami General Hospital, Sakamachi Hospital, Sanpoku Tokushukai Hospital, Sasaki Orthopaedic Clinic, Takahashi Orthopaedic Clinic, and Arakawa Chuo Clinic in Murakami City, Nakajo Chuo Hospital in Tainai City, Niigata Prefectural Shibata Hospital in Shibata City, and Tsuruoka Municipal Shonai Hospital in Tsuruoka City. Certification of long-term care insurance is investigated by the local governments. Dementia cases are tracked in the abovementioned hospitals and three additional medical institutions involved in neurology, including Murakami Hamanasu Hospital, Sano Clinic in Murakami City and Kurokawa Hospital in Tainai City. Information regarding disabilities is obtained from the long-term care insurance registry of local governments. Incident cases of cancers are followed with the cancer registry of Niigata Prefecture. Mortality and moving information are obtained from residency registration and death registration according to the Basic Residential Registry Law and Family Registry Law to calculate person-years of observation, because cases of death and moving out are censored.

### Self-administered questionnaire

The questionnaire was designed according to that used in the Japan Public Health Centre-based Prospective Study [11] (JPHC Study) and JPHC Study for the Next Generation (JPHC-NEXT Study) [12]. The questionnaire collected sociodemographic characteristics, family information, medical histories, body size, lifestyle, dietary intake [13]; physical and mental health (including chronic pain [14, 15]); and reproductive history (for women only). Details are provided in Table 1. Data management procedures were carried out based on the JPHC Studies [11, 12].

**Table 1:** Items of the self-administered questionnaire at baseline

Measures	
Sociodemographics	Age
	Sex
	Marital status
	Occupation
	Education
	Household income
Family information	Family structure
	Family disease histories
Medical history	Fractures
	Knee osteoarthritis
	Cancer
	Cardiovascular disease
	Cerebrovascular disease
	Other chronic diseases
	Histories of medication use
Body size	Current weight
	Weight in the previous year
	Weight at 20 years of age
	Birth weight
	Height
	Waist circumference
Lifestyle	Smoking status
	Alcohol consumption
	Tea and coffee consumption
	Physical activity
	Time spent outdoors
	Dietary habits
	Dietary and nutritional intake (intakes of energy, 53 nutrients, and 29 food groups) using a validated food frequency questionnaire [13]
	Sleep
Physical and mental health	Activities of daily living
	Chronic pain*
	General health status
	Quality of life
Reproductive history (for women only)	Menstrual history
	Pregnancy and birth histories
	Breastfeeding history
	History of hormone use
	History of toxaemia in pregnancy

*Pain was evaluated using the Short Form 36 verbal rating scale [14, 15]

We verified self-reported height and body weight using anthropometric data from health check examinations. Pearson’s and Spearman’s correlation coefficients between self-reported and measured data were 0.9160 and 0.9743 for male height, 0.9249 and 0.9753 for female height, 0.9490 and 0.9720 for male body weight, and 0.8737 and 0.9729 for female body weight, respectively (male *N* = 1752, female *N* = 2259, *P* < 0.0001 for all coefficients).

### Baseline blood collection and examination

Fasting or non-fasting blood specimens were drawn with EDTA-2Na-containing tubes (7 mL) during the day and immediately stored at 4 °C in all settings. Their plasma and buffy coat were obtained by centrifugation at 1613 × *g* for 10 min in the laboratory of Niigata University Division of Preventive Medicine and stored at − 80 °C until biochemical analysis. Plasma 25-hydroxyvitamin D (25[OH]D) concentrations were determined with the Liaison® 25OH Vitamin D Total Assay (DiaSorin Inc.; Stillwater, MN, USA). Intra- and inter-assay coefficient of variation (CV) values were 3.2–8.1% and 6.9–12.7%, respectively.

For laboratory analyses at health check examinations, routine blood tests were analysed at two certified clinical laboratories of the Shibata Comprehensive Health Care Service Centre (Niigata, Japan) and BML, Inc. (Tokyo, Japan). Routine standardisation and calibration tests were conducted at these facilities.

### Main outcome measures

The primary outcome of this study was osteoporotic fracture. The secondary outcomes were age-related diseases, including knee osteoarthritis, perception of chronic pain, dementia, disability (long-term care insurance use), and some cancers. Osteoporotic fracture was defined as a fracture caused by minimal trauma due to reduced bone strength [16] and typically included vertebral compression fracture, hip fracture, and other long-bone fractures. Knee osteoarthritis was diagnosed with the Kellgren-Lawrence grade scale. Chronic pain is defined as pain lasting more than 6 months [17], and its presence was determined with the self-administered questionnaire. Other outcomes included some cancers potentially involved in vitamin D status, such as colorectal and breast cancers.

### Future statistical plans

Cox’s proportional hazards model will be used to calculate hazard ratios (HRs) of most outcome variables in relation to levels of exposure. Regarding self-reported outcomes, including chronic pain, a logistic regression model will be used to calculate odds ratios (ORs). In the multivariate model, HRs and ORs will be adjusted for potential confounders.

### Sample size

Validity of the sample size of this study should be discussed. We predict that we will observe 193,914 person-years (14,364 participants × 90% × 15 years) in the 15-year follow-up. Regarding hip fracture, one of our major outcomes, 211 new cases will be observed during the 15-year follow-up (with a 90% follow-up rate), according to data from Fujiwara et al.’s 14-year cohort study on hip fracture [18]. With these constraints, we could detect relative risks of 1.7 for the highest quartile to the lowest quartile of a predictor, given an average estimated incidence of 0.016 (211/[14,364 × 90%]), 80% statistical power, and *α* = 0.05. When limited to participants who provide blood samples (*N* = 8497), we could detect relative risks of 2.0 for the highest quartile of plasma 25(OH)D, applying the same assumption. Because the incidences of the other outcomes in this study are higher than that of hip fracture, we could determine their risk factors with more statistical power.

### Participant characteristics

Mean ages of men and women were 59.2 (SD = 9.3, *N* = 6907) and 59.0 years (SD = 9.3, *N* = 7457), respectively. Demographic, physical, and lifestyle characteristics among the 14,364 participants are displayed by sex and blood sample status in Table 2. Past or current histories of self-reported musculoskeletal events or disorders are shown in Table 3. In women, prevalence of all histories increased significantly with age. In men, prevalence of history of spinal fracture, knee osteoarthritis, and chronic pain increased significantly with age, but prevalence of histories of forearm and hip fractures and fall did not. The mean plasma 25(OH)D concentration in all participants who provided blood samples was 50.3 nmol/L (SD = 18.2, *N* = 8497). Mean plasma 25(OH)D concentrations by sex, age group, and season of blood collection are shown in Table 4. Health check examination data by sex (*N* = 4014) are shown in Table 5.

**Table 2:** Characteristics (numbers) of 14,364 participants by sex and blood sample status

	Sex		Blood samples	
Characteristics	Men (*N* = 6907)	Women (*N* = 7457)	Present (*N* = 8497)	Absent (*N* = 5867)
Age group (years)	*P* = 0.1804		*P* < 0.0001	
≤ 49	1277 (18.5%)	1429 (19.2%)	1483 (17.5%)	1213 (20.7%)
50–59	1938 (28.1%)	2161 (29.0%)	2333 (27.5%)	1746 (29.8%)
60–69	2596 (37.6%)	2759 (37.0%)	3399 (40.0%)	1952 (33.3%)
≥ 70	1096 (15.9%)	1108 (14.9%)	1282 (15.1%)	956 (16.3%)
Education level	*P* < 0.0001		*P* < 0.0001	
Junior high school	1889 (28.2%)	2368 (32.6%)	2330 (28.0%)	1927 (34.2%)
High school	3597 (53.6%)	3360 (46.3%)	4258 (51.1%)	2699 (47.9%)
Junior college	614 (9.2%)	1338 (18.4%)	1263 (15.2%)	689 (12.2%)
University or higher	607 (9.0%)	194 (2.7%)	482 (5.8%)	319 (5.7%)
Household income (yen)	*P* < 0.0001		*P* = 0.0282	
0–2,990,000	2199 (33.6%)	2687 (41.2%)	1967 (38.3%)	2919 (36.8%)
3,000,000–5,990,000	2786 (42.6%)	2409 (36.9%)	1956 (38.1%)	3239 (40.8%)
6,000,000–8,990,000	1056 (16.1%)	944 (14.5%)	795 (15.5%)	1205 (15.2%)
9,000,000–11,990,000	305 (4.7%)	295 (4.5%)	253 (4.9%)	347 (4.4%)
≥12,000,000	190 (2.9%)	195 (3.0%)	160 (3.1%)	225 (2.8%)
Activities of daily living	*P* = 0.0455		*P* < 0.0001	
No disability	6369 (93.5%)	6798 (92.3%)	7968 (94.1%)	5199 (91.1%)
Some disability, but able to go out alone	399 (5.9%)	520 (7.1%)	481 (5.7%)	438 (7.7%)
Living by oneself indoors, but need help outdoors	25 (0.4%)	26 (0.4%)	11 (0.1%)	40 (0.7%)
Need help indoors	4 (0.1%)	2 (0.0%)	0 (0.0%)	6 (0.1%)
Bedridden	14 (0.2%)	19 (0.3%)	6 (0.1%)	27 (0.5%)
Body mass index	*P* < 0.0001		*P* < 0.0001	
< 18.5	206 (3.0%)	492 (6.6%)	400 (4.7%)	298 (5.2%)
18.5–24.9	4614 (67.2%)	5313 (71.7%)	6040 (71.2%)	3887 (67.2%)
25.0–29.9	1810 (26.4%)	1341 (18.1%)	1791 (21.1%)	1360 (23.5%)
≥ 30.0	233 (3.4%)	261 (3.5%)	256 (3.0%)	238 (4.1%)
Smoking (cigarettes/day)	*P* < 0.0001		*P* < 0.0001	
Non-smoker	1244 (18.1%)	6335 (85.5%)	4868 (57.4%)	2711 (46.8%)
Past smoker	3338 (48.6%)	548 (7.4%)	2312 (27.3%)	1574 (27.2%)
1–19	824 (12.0%)	394 (5.3%)	582 (6.9%)	636 (11.0%)
≥ 20	1462 (21.3%)	130 (1.8%)	721 (8.5%)	871 (15.0%)
Alcohol consumption (grams of ethanol/week)	*P* < 0.0001		*P* < 0.0001	
None or rarely	1333 (19.4%)	4899 (66.0%)	3715 (43.8%)	2517 (43.3%)
1–149	1730 (25.2%)	2009 (27.1%)	2419 (28.5%)	1320 (22.7%)
150–299	1335 (19.4%)	271 (3.7%)	935 (11.0%)	671 (11.6%)
300–449	1213 (17.6%)	149 (2.0%)	753 (8.9%)	609 (10.5%)
≥ 450	1265 (18.4%)	91 (1.2%)	666 (7.9%)	690 (11.9%)
Sex			*P* < 0.0001	
Men	–	–	3710 (43.7%)	3197 (54.5%)
Women	–	–	4787 (56.3%)	2670 (45.5%)

*P* values were calculated by the *χ*^2^ test between men and women and between those with and without blood samples. Missing values are generated except for “age group” and “sex”

**Table 3:** Past or current histories of self-reported musculoskeletal events or disorders in 14,364 participants by sex and age groups

	Men					Women				
Musculoskeletal disorders	≤ 49 years old	50–59 years old	60–69 years old	≥ 70 years old	*P* for trend	≤ 49 years old	50–59 years old	60–69 years old	≥ 70 years old	*P* for trend
Past history of fracture*of										
Lumbar region	3/1272	4/1933	12/2593	12/1093	0.0018	1/1423	4/2154	12/2752	9/1102	0.0011
	(0.2%)	(0.2%)	(0.5%)	(1.1%)		(0.1%)	(0.2%)	(0.4%)	(0.8%)	
Forearm	37/1272	71/1933	76/2593	22/1093	0.1107	20/1423	35/2154	89/2752	51/1102	< 0.0001
	(2.9%)	(3.7%)	(2.9%)	(2.0%)		(1.4%)	(1.6%)	(3.2%)	(4.6%)	
Hip	6/1272	6/1933	5/2593	4/1093	0.4062	0/1423	1/2154	5/2752	3/1102	0.0272
	(0.5%)	(0.3%)	(0.2%)	(0.4%)		(0.0%)	(0.0%)	(0.2%)	(0.3%)	
Past history of fall^†^	272/1234	368/1875	496/2535	248/1064	0.6960	212/1395	383/2114	497/2700	244/1072	< 0.0001
	(22.0%)	(19.6%)	(19.6%)	(23.3%)		(15.2%)	(18.1%)	(18.4%)	(22.8%)	
Current knee osteoarthritis	13/1265	52/1913	204/2576	131/1078	< 0.0001	31/1417	158/2145	397/2735	273/1091	< 0.0001
	(1.0%)	(2.7%)	(7.9%)	(12.2%)		(2.2%)	(7.4%)	(14.5%)	(25.0%)	
Current chronic pain^‡^	389/1265	631/1913	988/2576	436/1078	< 0.0001	442/1417	782/2145	1107/2735	527/1091	< 0.0001
	(30.8%)	(33.0%)	(38.4%)	(40.5%)		(31.2%)	(36.5%)	(40.5%)	(48.3%)	

*Subjects were asked to report fractures that were not caused by high energy trauma, such as a motor vehicle accident, fall from a higher level than a standing height, or occupational accident

^†^Subjects were asked to report falls from a standing height or less

^‡^Subjects were asked to report pain lasting for more than 6 months in any part of the body

**Table 4:** Mean plasma 25-hydroxyvitamin D concentrations and standard deviations (SDs) by sex, age group, and season in 8497 participants providing blood samples

		25-hydroxyvitamin D (nmol/L)		
	*N*	Mean	SD	*P* value*
Sex				
Men	3710	56.5	18.4	< 0.0001
Women	4787	45.4	16.5	
Age group (years)				
< 49	1489	42.9	17.6	Reference
50–59	2345	47.7	17.7	< 0.0001
60–69	3400	53.7	17.9	< 0.0001
≥ 70	1263	54.5	17.0	< 0.0001
Season of blood collection				
Spring (Mar–May)	1905	44.9	17.4	Reference
Summer (Jun–Aug)	3890	50.8	17.6	< 0.0001
Autumn (Sep–Nov)	2375	53.9	18.6	< 0.0001
Winter (Dec–Feb)	327	48.8	19.5	0.0047

Multiply the values by 0.4 to convert nmol/L to ng/mL for 25-hydroxyvitamin D

*T test was used to analyse sex, and Dunnett’s test was used for the other variables

**Table 5:** Results of health check examinations (*N* = 4014)

	Men		Women	
Characteristics	*N*	Mean (SD)	*N*	Mean (SD)
Body mass index (kg/m^2^)	1753	23.6 (3.1)	2261	22.6 (3.3)
Waist circumference (cm)	1695	84.7 (8.5)	2237	80.5 (9.1)
Systolic blood pressure (mmHg)	1753	129.6 (17.3)	2261	123.5 (16.8)
Diastolic blood pressure (mmHg)	1753	79.3 (10.7)	2261	73 (10.3)
Serum HDL cholesterol (mg/dL)	1712	56 (14.7)	2247	63.7 (14.5)
Serum LDL cholesterol (mg/dL)	1712	109.4 (28.7)	2247	118.5 (27.4)
Serum triglyceride (mg/dL)	1712	151.1 (117.5)	2247	114.8 (73.4)
Serum ALT (U/L)	1712	24.5 (13.9)	2247	18.6 (10)
Serum AST (U/L)	1712	26.4 (11.9)	2247	22.3 (7)
Serum γ-GTP (U/L)	1712	49.6 (64.8)	2247	21.3 (19.5)
Blood sugar (mg/dL)	1474	112.2 (31.6)	1820	104.3 (26.8)
Blood HbA1c (%)	1441	5.7 (0.6)	1870	5.6 (0.4)
Blood haemoglobin (g/dL)	1691	14.5 (1.3)	2018	12.9 (1.2)
Red blood cell count	1691	463 (42)	2018	433 (35)

*ALT* alanine aminotransferase, *AST* aspartate aminotransferase, *γ-GTP* gamma-glutamyltransferase, *HbA1c* glycated haemoglobin

## Discussion

A number of population-based cohort studies to determine risk factors for osteoporotic fracture have been conducted in European and North American countries [19]. Among them, large-scale studies (*n* > 5000) include the CaMos (*N* = 5143, ≥ 25 years old) [20], EPIC-Norfolk (*N* = 14,824, 42–82 years old) [21], EPIDOS (*N* = 7598, ≥ 75 years old) [22], OSTPRE (*N* = 12,191, 47–56 years old) [23], PERF (*N* = 5564, 45–70 years old) [24], Rotterdam (*N* = 14,926, ≥ 45 years old) [25], and SOF (*N* = 9516, ≥ 65 years old) [26] studies. In Japan, the Fujiwara-kyo Study (*N* = 4427, ≥ 65 years old) [27] is an ongoing cohort study on osteoporotic fractures. The Murakami Cohort Study is considered one of the largest studies on osteoporotic fractures, comparable to previous studies.

We have established a population-based cohort study on musculoskeletal and other age-related diseases with a sufficient sample size and plan to follow our population for 15 years. We anticipate that we will be able to clarify the association between vitamin D status and multiple disease outcomes in a Japanese population. The present study is one of the largest cohort studies to clarify the effects of vitamin D in an Asian population. One strength of this cohort study is related to the geographical features of the Murakami region. As it is surrounded by mountains and sea, this region has high rates of self-sufficient medical care, namely, its self-sufficient rates for the three fatal diseases in Murakami City have been reported to be 68.5% for cancer, 91.1% for cerebrovascular disease, 80.8% for cardiovascular disease, and 81.4% for a total of the three diseases [28]. Self-sufficiency for medical care of unfatal diseases, such as musculoskeletal diseases, is considered to be equal to or higher than that for fatal diseases. Therefore, we should be able to identify most cases of our targeted diseases.

This study has some potential limitations. First, although a large number of people participated, the participation rate was not very high (41.3%). In addition, no information on medication use was collected from participants. Therefore, our results may not accurately reflect the entire population of the study area. Second, generalisation of our results should be made with caution. The Murakami region includes medium- and small-sized local governments. Therefore, while our results can be generalised to regions with similar-sized populations, they may not apply to regions with larger local governments or metropolitan governments (e.g., Tokyo). Moreover, lifestyles differ between the Murakami region and metropolitan regions. For instance, many elderly living in medium or small communities in Japan retain old, traditional lifestyles, which also include particular dietary habits. Finally, in some fracture studies, bone mineral density (BMD) is measured to evaluate individual bone mass, because low BMD, i.e., osteopenia or osteoporosis, is established as a clinical risk factor for osteoporotic fracture. For this reason, the present study does not contribute to the prevention of osteoporosis or to the secondary prevention of osteoporotic fracture.

## Abbreviations

25(OH)D: 25-Hydroxyvitamin D
ALT: Alanine aminotransferase
AST: Aspartate aminotransferase
BMD: Bone mineral density
CaMos: Canadian Multicentre Osteoporosis Study
CV: Coefficient of variation
EDTA-2Na: Ethylenediaminetetraacetic acid disodium salt,2-hydrate
EPIC-Norfolk: Norfolk cohort of the European Prospective Investigation into Cancer
EPIDOS: EPIDemiologie de l’OStéoporose
HbA1c: Glycated haemoglobin
HDL: High-density lipoprotein
HR: Hazard ratio
JPHC Study: Japan Public Health Centre-based Prospective Study
LDL: Low-density lipoprotein
OR: Odds ratio
OSTPRE: Osteoporosis Risk Factor and Prevention
PERF: Prospective Epidemiological Risk Factors
SD: Standard deviation
SOF: Study of Osteoporotic Fractures
γ-GTP: Gamma-glutamyltransferase
